# Lurbinectedin in treating CNS metastases from small cell neuroendocrine prostate cancer—a case report

**DOI:** 10.3389/fonc.2026.1840883

**Published:** 2026-07-09

**Authors:** Avani Gunuganti, Vedin Barve, William J. Kelly, Vijay Gunuganti, Andrew Brenner

**Affiliations:** 1 Rice University, Houston, TX, United States; 2 Department of Medicine San Antonio, The University of Texas Health Science Center, San Antonio, TX, United States; 3 Texas Oncology, San Antonio, TX, United States

**Keywords:** prostate cancer, CNS metastasis, lurbinectedin, small cell lung cancer, neuroendocrine

## Abstract

**Background:**

*De novo* neuroendocrine prostate cancer is an aggressive, androgen receptor-independent subtype of prostate cancer with poor prognosis. Central nervous system (CNS) metastases from *de novo* neuroendocrine prostate tumors are rare, and evidence guiding treatment is limited. Lurbinectedin, approved for small cell lung cancer (SCLC), has shown activity in neuroendocrine tumors, though CNS-specific data remain sparse.

**Case presentation:**

A 57-year-old man presenting with tenesmus, dyschezia, and urinary retention was diagnosed with high-grade neuroendocrine prostate carcinoma with low prostate-specific antigen (PSA) and high proliferative index. Genomic profiling revealed alterations in ATM and BRCA2 and was negative for Rb1 and TP53. He achieved complete systemic response following platinum-based chemoradiotherapy and immunotherapy but later developed multifocal brain metastases with suspected leptomeningeal involvement.

**Intervention and outcomes:**

After progression on whole-brain radiotherapy and topotecan, lurbinectedin was initiated. Imaging demonstrated marked intracranial response, including resolution and reduction of multiple lesions. Disease progression occurred after six cycles.

**Discussion:**

This case underscores similarities between neuroendocrine prostate carcinoma and SCLC, supporting the use of SCLC-based therapies. The observed response suggests that lurbinectedin may have clinically meaningful CNS activity, even after failure of standard CNS-directed treatments.

**Conclusion:**

Lurbinectedin may be a therapeutic option in CNS-dominant neuroendocrine prostate carcinomas. Further studies are needed to better define its intracranial efficacy and durability of response.

## Introduction

Prostate cancer is the second most frequently diagnosed cancer and a leading cause of cancer mortality worldwide for men (1). While most tumors are androgen-sensitive acinic adenocarcinomas and respond to androgen deprivation therapy (ADT), *de novo* neuroendocrine prostate carcinoma (NEPC) is rare, aggressive, and associated with visceral metastases, elevated serum neuroendocrine markers (chromogranin A and neuron-specific enolase), and a low serum prostate-specific antigen (PSA) relative to disease burden (2, 3). Of note is that *de novo* NEPC is different from the much more common treatment-emergent NEPC (t-NEPC), which is described as neuroendocrine transformation of a prostatic adenocarcinoma after ADT (1).

Limited research exists on the treatment of central nervous system (CNS) metastases from *de novo* NEPC, largely due to the overall rarity of *de novo* NEPC. Several large series report incidences well below 2% among all prostate cancer (4). Within this already uncommon population, CNS involvement from prostate cancer of small cell/neuroendocrine origin is particularly rare, with only a small minority of reported CNS cases demonstrating small cell or neuroendocrine histology (5, 6).

Lurbinectedin is an emerging therapy of interest for the treatment of neuroendocrine tumors. Lurbinectedin is a selective DNA binding agent with preferential binding to GC-rich sequences, and an inhibitor of oncogenic transcription by blocking RNA polymerase II. Lurbinectedin is currently Food and Drug Administration (FDA) approved for adults with metastatic small cell lung cancer (SCLC) after progression on platinum-based therapies as well in patients with extensive-stage SCLC (7). Despite its promising results in several studies, lurbinectedin has not been approved for use in other neuroendocrine tumors, and relevant literature regarding the use of lurbinectedin in treating CNS metastases from primary neuroendocrine tumors remains sparse.

This case is notable for the rare occurrence of NEPC with extensive CNS metastases and probable leptomeningeal involvement. It further highlights a marked intracranial response to lurbinectedin following progression on both whole-brain radiotherapy and known CNS-penetrant chemotherapy, an area where clinical evidence remains limited.

## Case presentation

### Presentation and clinical findings

The patient was a 57-year-old man with a history of diabetes, hypertension, and hyperlipidemia who presented initially to gastroenterology with tenesmus and dyschezia. Magnetic resonance imaging (MRI) of the lumbar spine on Day 0 showed only degenerative changes. CT abdomen pelvis (Month 2) showed an enlarged heterogeneous prostate gland with posterior extension. He also endorsed urinary retention and slow stream of urine for several months. Evaluation by urology (Month 3) revealed an enlarged, tender, and nodular prostate. PSA at that time was 0.26 ng/mL.

### Pathological diagnosis

The patient underwent prostate biopsy (Month 3). Pathology was read as high-grade neuroendocrine carcinoma with 40%–100% involvement in 10 of 16 cores. Specifically, immunostains were positive for INSM1, synaptophysin, and cytokeratin. The stains were negative for cyclin D1 and chromogranin. Staining for NKX 3.1 was also negative, which excluded acinic adenocarcinoma from the differential. Ki-67 read as “very high proliferation”. TEMPUS XT profiling revealed alterations in ATM (c.4909+G>T splice region variant with LOF), BRAC2 (copy number loss), and CREBBP (p.G138sfs with LOF), a tumor mutation burden of 2.1 m/MB, and variants of unknown significance (VUS) in ERCC6, CYLD, IFNGR1, and BRCA1. NGS sequencing, however, did not reveal molecular alterations in Rb1 and TP53. PD-L1 (22C3) showed TPS <1% and CPS <1. Liquid biopsy from peripheral blood (Month 4) showed alterations in PIK3CA (p.P104R with GOF) and BRCA2 (copy number loss) as well as VUS in BRCA1, AXIN2, and POLE.

F-18 PET (Month 4) showed abnormal appearance and uptake in the prostate gland with soft tissue avidity extending beyond capsule to involve the rectum, as well as enlargement and avidity in pelvic lymph nodes with avid sclerotic lesions in the pelvis and L1 vertebra. This patient was not presented to a tumor board.

### Therapeutic intervention

Initial therapy utilized a multidisciplinary approach from both medical oncology and radiation oncology to provide both palliative and curative therapy. The patient received external radiation to the prostate at a 60-Gray (Gy) dose, concurrent with seven cycles of weekly carboplatin and paclitaxel (Months 4 and 5). Following completion of this, the patient received five cycles of carboplatin plus etoposide and atezolizumab (Months 6–9) with G-CSF support followed by one cycle of maintenance atezolizumab (Month 10). This regimen was selected given that the patient presented with visceral metastases, bulky disease, and a low PSA and was considered “medically fit”. F-18 PET (Month 8) during the combination therapy showed resolution of abnormal tracer activity and decreased size of the prostate gland as well as resolution of hypermetabolic nodal and osseous metastases. CT TAP (Month 13) after atezolizumab showed no evidence of tumor.

Unfortunately, the patient suffered seizure (Month 17) with aphasia, tonic limbs, and loss of consciousness. MRI brain (Month 17) showed numerous subcentimeter enhancing lesions scattered throughout the brain. CT TAP (Month 17) showed only urinary bladder wall thickening. LP showed no cancer cells.

The patient completed whole-brain radiotherapy (Months 17 and 18). MRI brain (Month 22) showed dural-based, extra-axial enhancing solid mass along right middle cranial fossa floor (meningioma vs. metastasis). F-18 PET (Month 24) continued to show no evidence of recurrent or metastatic disease. However, MRI brain (Month 25) showed interval progression from the MRI brain on Month 22 with an enlarged, enhancing inferior right temporal lesion (0.8 × 1 cm from 0.5 cm), as well as multiple new cerebral cortical enhancing lesions and small surface enhancement of cerebellum consistent with leptomeningeal involvement. MRI spine (Month 26) showed no evidence of neoplastic disease.

The patient subsequently underwent fluoroscopic-guided lumbar puncture (Month 26), which showed only mild pleocytosis with no malignant cells. MRI brain (Month 27, **Figure 1A**) showed interval progression of intracranial metastatic disease with increased size and number of numerous supratentorial and infratentorial metastasis including cortical surface and adjacent sulci involvement suspicious for leptomeningeal involvement. At this point in time, radiation oncology suggested that further radiation therapy may lead to significant neurological decline and advised against further radiation. The patient was offered a referral for potential clinical trials, but the patient declined that as well.

**Figure 1:**
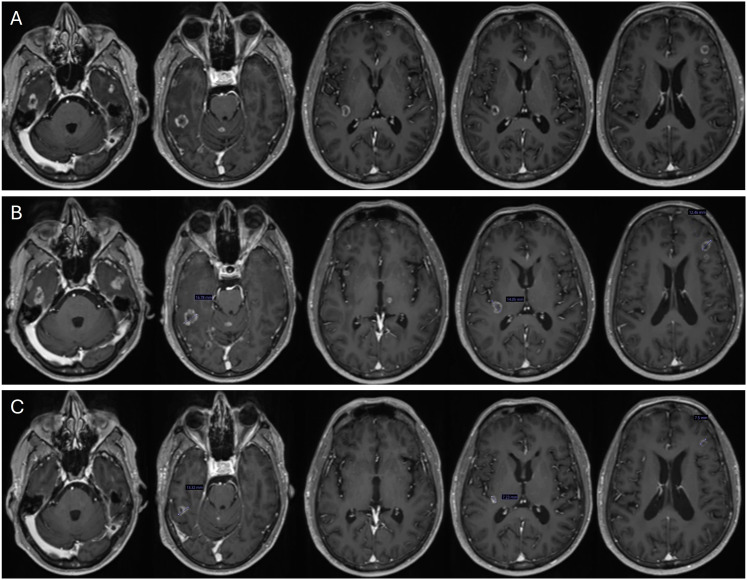
**(A)** MRI Brain at month 27 showing intracranial metastatic disease including numerous supratentorial and infratentorial metastases involving cortical surface and adjacent sulci involvement suspicious for leptomeningeal involvement; **(B)** MRI Brain at month 29 showing baseline multifocal-rim enhancing parenchymal and probable radiographic leptomeningeal metastases prior to the start of lurbinectedin; **(C)** MRI Brain at month 32 showing marked decrease in several intracranial lesions including complete resolution of some metastases after starting lurbinectedin.

The patient then received three cycles of topotecan (Months 28–30). MRI brain (Month 29, **Figure 1B**) during topotecan treatment showed mild enlargement of several of the rim-enhancing brain metastases, suggesting treatment resistance. The patient was then started on lurbinectedin 3.2 mg/m^2^ IV q21 days (Month 31).

### Follow-up and outcomes

MRI brain (Month 32, **Figure 1C**) showed interval resolution of multiple lesions with the seven remaining lesions showing a significant decrease in size when compared to the MRI brain on Month 28 (**Figure 1B**). This included a ring-enhancing left frontal (0.7 from 1.4 cm), ring-enhancing medial left frontoparietal (0.6 from 0.9 cm), punctate-enhancing left occipital (0.3 from 0.8 cm), enhancing left anterior temporal (0.7 from 1.4 cm), ring-enhancing posterior right insula (0.7 from 1.2 cm), ring-enhancing posterior right temporal (1.1 from 1.7 cm), and punctate-enhancing superior vermis (0.3 from 1.7 cm). During lurbinectedin treatment, the patient experienced increased fatigue and hypersomnia, constipation, sinus pain, and grade 1 anemia, lymphopenia, and neutropenia. Constipation was managed with polyethylene glycol 3350. Shortly after cycle 6 of lurbinectedin, MRI brain indicated radiographic progression. Unfortunately, the patient is unable to provide their perspective on the treatment provided. At the time of the development of this manuscript, the patient had entered hospice.

## Discussion

The patient is a 57-year-old man with *de novo* NEPC who achieved excellent initial systemic control from prostate chemoradiotherapy with weekly carboplatin/paclitaxel followed by carboplatin–etoposide plus atezolizumab and maintenance atezolizumab but ultimately developed multifocal intracranial metastases with leptomeningeal involvement. Lurbinectedin was subsequently initiated after whole-brain radiotherapy and topotecan, and was associated with a marked intracranial radiographic response, with resolution of several lesions and substantial reduction in the size of remaining metastases on follow-up MRI after six cycles.

Lurbinectedin has shown promising results in the treatment of SCLC. In a single-arm phase II trial (NCT02454972), lurbinectedin achieved an overall response rate of 35.2% with a median progression-free survival (PFS) of 3.5 months and an overall survival (OS) of 9.3 months in relapsed SCLC (8). Additionally, the combination of lurbinectedin with atezolizumab significantly improved PFS (5.4 vs. 2.1 months; HR 0.54) and OS (13.2 vs. 10.6 months; HR 0.73) compared with atezolizumab alone in extensive-stage disease (9).

Beyond SCLC, lurbinectedin has shown activity across multiple neuroendocrine and solid tumors. In a phase I basket study, objective tumor shrinkage was observed in 55.9% of evaluable patients, with antitumor activity noticeably elevated in SCLC, soft tissue sarcoma, endometrial carcinoma, glioblastoma, and pancreatic adenocarcinoma when treated with a combination of lurbinectedin + irinotecan (10). Similarly, a retrospective series of 18 patients with small cell and NEPC conducted by Meyer et al. reported a 56% clinical benefit rate, with a median PFS of 3.35 months and a median OS of 6.01 months (11). Despite these advances, data on lurbinectedin in *de novo* NEPC remain limited.

The potential role of lurbinectedin in non-SCLC neuroendocrine tumors may be explained by the shared molecular features between these malignancies and SCLC tumors. SCLC is characterized by high proliferative indices (elevated Ki-67), frequent loss-of-function alterations in key tumor suppressor genes such as TP53 and RB1, and alterations in DNA damage repair genes including ATM and BRCA1/2 (12–14). While our patient’s NGS demonstrated an absence of TP53 and RB1 alterations, the absence of any acinic adenocarcinoma on tissue histology supports the notion that this tumor is likely *de novo* NEPC. Furthermore, the tumor’s shared molecular features with SCLC tumors (e.g., mutations in ATM, BRCA1/2, and a high proliferative index) supports the extrapolation of SCLC-approved therapeutic strategies to NEPCs once standard treatment options have been exhausted.

This biological rationale is further supported by the patient’s clinical course, particularly within the CNS. CNS metastases from small cell and neuroendocrine tumors are typically radiosensitive, and whole-brain chemoradiotherapy is generally effective in inducing at least temporary responses (15). However, in this case, the patient showed CNS progression after whole-brain radiotherapy and after a course of topotecan, a treatment with known blood–brain penetration and established use in CNS and SCLC settings. Notably, treatment with lurbinectedin produced a substantial radiographic response after failure of these modalities, suggesting meaningful CNS penetration and clinically significant intracranial antitumor activity. Evidence regarding the CNS activity of lurbinectedin remains limited; in the series by Meyer et al., three patients had CNS metastases at treatment initiation, two of whom achieved response or stable disease for at least 6 months (11). NCT02454972 specifically excluded patients with known CNS involvement from their study; thus, research has yet to be done regarding lurbinectedin as a therapeutic option for patients with CNS-predominant disease who have progressed on conventional CNS-penetrant therapies. The rarity of CNS metastases from primary NEPC tumors, along with the limited literature regarding the treatment of these cases, underscores the clinical relevance of the present case, which provides one of the few detailed examples to inform management when small cell neuroendocrine prostate cancers present with extensive intracranial and probable leptomeningeal metastases.

Interpretation of this case is limited by several factors. The relatively short follow-up in this case restricts assessments of durability. CNS lesions were not biopsied, leaving some diagnostic uncertainty. Finally, prior whole-brain radiotherapy could have altered blood–brain barrier integrity, meaning observed CNS activity upon the administration of lurbinectedin could reflect radiation-facilitated drug penetration rather than intrinsic CNS efficacy, limiting generalizability.

Despite these limitations, this case highlights several key clinical insights. It suggests that lurbinectedin may have meaningful CNS activity and can be useful in other tumors with neuroendocrine features. For clinicians managing advanced prostate cancer with CNS involvement, this case portrays the importance of early recognition of NEPC, given its unique clinical features, reassessment of molecular profiles for SCLC-like features as the disease evolves, and consideration of lurbinectedin-based strategies when CNS-dominant progression occurs despite standard systemic and radiation therapies. Practical takeaways include involving neuro-oncology and radiation oncology early when neurologic symptoms arise, and discussing SCLC-derived options, such as lurbinectedin, in multidisciplinary tumor boards for patients with CNS-predominant NEPC who have exhausted conventional lines of therapy.

Note: A summary table of the cited studies pertaining to the use of lurbinectedin can be found in **Table 1**. A timeline of relevant events can be found in **Table 2**.

**Table 1:** A summary of studies on the use of lurbinectedin in small cell cancer and other neuroendocrine cancers.

Author(s)	Year	Study type/Population	Key findings
Trigo J, Subbiah V, Besse B, et al. (8)	2020	Single-arm phase II trial in relapsed small cell lung cancer	Lurbinectedin achieved an ORR of 35.2%, median PFS 35 months, median OS 9.3 months in relapsed patients with SCLC.
Paz-Ares L, Borghaei H, Liu S V, et al. (9)	2025	IMforte: Phase III multicenter study of atezolizumab + lurbinectedin in extensive-stage SCLC	Atezolizumab + lurbinectedin combination improved outcomes compared to atezolizumab alone. PFS 5.4 vs. 2.1 months (HR 0.54) and OS 13.2 months vs. 10.6 months (HR 0.73)
Falcón A, Ponce S, Cote GM, et al. (10)	2025	Phase I study evaluating combination of lurbinectedin + irinotecan in patients with relapsed advanced solid tumors	Objective tumor shrinkage was seen in 55.9% of patients with a manageable and predictable toxicity profile. Antitumor activity was notably higher in SCLC, synovial sarcoma, endometrial carcinoma, glioblastoma, and pancreatic adenocarcinoma
Meyer H, Sunkara R, Rothmann E, et al. (11)	2024	Retrospective series of 18 patients with small cell/neuroendocrine prostate carcinoma treated with lurbinectedin.	56% clinical benefit rate, median PFS 3.35 months, and median OS 6.01 months across all patients. Among 3 patients with CNS metastases, 2 achieved response or stable disease for at least 6 months.

**Table 2:** Timeline

Time	Events
Months 1–3	Presentation and clinical findings: Patient presented with tenesmus, dyschezia, and urinary symptoms. MRI lumbar spine showed degenerative changes; CT abdomen/pelvis showed enlarged heterogeneous prostate gland; PSA was 0.26 ng/mL. Urology evaluation revealed enlarged, tender, nodular prostate.
Month 4	Pathological diagnosis: Prostate biopsy confirmed high-grade neuroendocrine carcinoma with 40%–100% involvement; immunostains positive for INSM1, synaptophysin, cytokeratin; Ki-67 very high. Molecular profiling revealed alterations in ATM, BRCA2, CREBBP, and others; F-18 PET scan showed prostate lesion with extracapsular extension, pelvic lymphadenopathy, and bone metastases.
Months 4–34	Therapeutic intervention: Therapeutic interventions included the following: external radiation (60 Gy) to prostate with concurrent weekly carboplatin and paclitaxel (seven cycles), followed by five cycles carboplatin, etoposide, and atezolizumab with G-CSF support, then maintenance atezolizumab. Initial imaging showed resolution of nodal and osseous metastases. Patients developed seizures and brain metastases, and received whole-brain radiotherapy. CNS progression followed with multiple enhancing brain lesions and probable leptomeningeal involvement. Subsequent treatment with topotecan showed mild progression. Lurbinectedin initiated (3.2 mg/m² IV every 21 days), resulting in significant intracranial radiographic response.
Months 32–34	Follow-up and outcomes: MRI brain on Month 32 showed resolution or marked reduction in size of multiple brain metastases; however, radiographic progression was noted shortly after cycle 6 of lurbinectedin (Month 34).

## Conclusion

This case illustrates the potential efficacy of lurbinectedin in treating CNS-dominant metastases from neuroendocrine prostate cancer, even after progression on whole-brain radiotherapy and CNS-penetrant chemotherapy. The observed intracranial radiographic response, along with the molecular parallels between NEPC and SCLC, supports the rationale for extrapolating SCLC-approved therapies to this rare and aggressive prostate cancer subtype. While interpretation is limited by the short follow-up, the absence of tissue confirmation for CNS lesions, and potential effects of prior radiotherapy on blood–brain barrier integrity, the case highlights actionable insights for clinical practice. It underscores the importance of early recognition of NEPC, systemic molecular reassessment for SCLC-like features, and consideration of lurbinectedin-based strategies in patients with CNS-predominant progression who have exhausted conventional systemic and radiation options. Further studies are warranted to clarify the intracranial activity, pharmacokinetics, and durability of lurbinectedin in this population, as well as to inform evidence-based management of CNS metastases in NEPC.

## Data Availability

The original contributions presented in the study are included in the article. Further inquiries can be directed to the corresponding author.
